# Prevalence and Associated Risk Factors of Gastrointestinal Parasite Infections among Meat Goats in Khon Kaen Thailand

**DOI:** 10.1155/2024/3267028

**Published:** 2024-09-03

**Authors:** Sarinya Rerkyusuke, Sawarin Lerk-u-suke, Raktham Mektrirat, Anuwat Wiratsudakul, Prapan Kanjampa, Saikam Chaimongkol, Nattaya Phanmanee, Miranda Visuddhangkoon, Panicha Pattayawongdecha, Nanticha Piyapattanakon, Pongpatchara Krajaipan, Pitchapa Sutamwirat

**Affiliations:** ^1^ Division of Livestock Medicine Faculty of Veterinary Medicine Khon Kaen University, Khon Kaen 40002, Thailand; ^2^ KKU Research Program Khon Kaen University, Khon Kaen 40002, Thailand; ^3^ Department of Geographic Information Science School of Information and Communication Technology University of Phayao, Phayao 56000, Thailand; ^4^ Research Unit of Spatial Innovation Development School of Information and Communication Technology University of Phayao, Phayao 56000, Thailand; ^5^ Veterinary Academic Office Faculty of Veterinary Medicine Chiang Mai University, Muang, Chiang Mai 50100, Thailand; ^6^ Research Center for Veterinary Biosciences and Veterinary Public Health Faculty of Veterinary Medicine Chiang Mai University, Chiang Mai 50100, Thailand; ^7^ Department of Clinical Sciences and Public Health and the Monitoring and Surveillance Center for Zoonotic Diseases in Wildlife and Exotic Animals Faculty of Veterinary Science Mahidol University, Nakhon Pathom 73170, Thailand; ^8^ Laboratory Service and Laboratory Animal Unit Faculty of Veterinary Medicine Khon Kaen University, Khon Kaen 40002, Thailand; ^9^ Veterinary Diagnostic Laboratory Faculty of Veterinary Medicine Khon Kaen University, Khon Kaen 40002, Thailand; ^10^ Faculty of Veterinary Medicine Khon Kaen University, Khon Kaen 40002, Thailand

## Abstract

This study investigated the epidemiology of gastrointestinal (GI) parasite infections among 42 meat goat herds in Khon Kaen, Northeast Thailand, based on 913 fecal samples. The predominant parasites identified in the herd were strongyle (100.0%, 42/42), *Trichuris* spp. (73.8%, 31/42), *Eimeria* spp. (66.7%, 28/42), *Moniezia* spp. (64.3%, 27/42), *Strongyloides* spp. (38.1%, 16/42), and *Paramphistomum* spp. (7.1%, 3/42). Coinfection with at least two GI parasites was observed in 90.4% of the herds. Molecular analysis confirmed *Haemonchus contortus* and *Trichostrongylus* spp. as the strongyle species. The study explored parasite prevalence among animals, finding significant correlations with season, sex, age, and breed. Notably, the wet season showed increased strongyle and *Eimeria* spp. infections. Female animals had higher odds of strongyle infection, while younger animals (less than 1 year) were more susceptible. Conversely, animals aged over 1 year were more likely to be positive for *Trichuris* spp., *Moniezia* spp., and *Eimeria* spp. infections. Female animals exhibited poor body condition scores (BCS) and anemia, as indicated by the FAMACHA score and packed cell volume (PCV) levels. Correlations between age, clinical signs, hematological parameters, biochemistry, and GI parasite burdens were investigated, revealing significant associations. These findings emphasize the need for tailored intervention strategies considering seasonal variations, age, and sex differences for effective GI parasite control in meat goats. Prioritizing animals exhibiting poor BCS and elevated FAMACHA score is imperative to mitigate the deleterious impacts of GI parasitic infections on health and productivity.

## 1. Introduction

Gastrointestinal (GI) parasite infections are a major health concern for small ruminants worldwide, causing significant losses in productivity, economy, and welfare [1–5]. Studies have estimated that helminth infections cost approximately €1.8 billion annually in Europe alone, with most of the losses attributed to decreased production [2]. GI parasites include nematodes, cestodes, trematodes, and protozoa, and they cause symptoms, such as reduced feed intake, slow growth, anemia, diarrhea, rough hair, and poor BCS, which can lead to higher mortality rates in herds. In Thailand, the small ruminant population, primarily consisting of 1.5 million goats and 0.1 million sheep, has been steadily increasing by approximately 28% per year [6, 7]. Farmers typically implement deworming programs every one to six months using drugs like albendazole, ivermectin, and levamisole. However, due to variations in GI parasite infections, tailored treatment, control, and prevention strategies are necessary. This study aims to document the epidemiology of GI parasite-infected meat goats in tropical zones like Khon Kaen, Thailand, and establish associations between factors and clinical signs based on laboratory findings. The information obtained from this study could help develop effective management programs to address challenges related to GI parasites.

## 2. Materials and Methods

### 2.1. Study Area and Design

The study was conducted from March to December 2021 in Khon Kaen province, situated at coordinates 16°26′48.16″N and 102°49′58.8″E with an altitude of 187 meters above sea level. The area experiences a range of annual temperatures, from 13.9°C to 39.6°C (minimum to maximum), and annual rainfall averaging between 0.0 and 9.38 mm. Relative humidity fluctuates between 21% and 99% [8]. The seasons are classified into two primary categories: the wet or monsoon season, which spans from May to October and is characterized by increased rainfall and higher humidity levels, and the dry season. The dry season is further subdivided into two distinct periods: the summer season, occurring from March to April, and the winter season, occurring from November to February. Both of these subseasons are marked by lower humidity levels.

**Figure 1:**
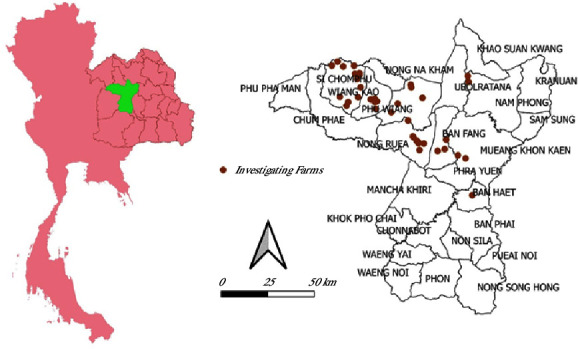
The geographic distribution of meat goat herds in Khon Kaen, Northeast Thailand.

A total of 42 meat goat herds across 8 districts in Khon Kaen province were included in the study, comprising Ban Fang (3), Ban Haet (1), Muang (2), Nam Phong (2), Nong Ruea (9), Phu Wiang (5), Si Chomphu (13), and Wiang Kao (7) districts (Figure 1). Additionally, 1,868 animals underwent physical examinations to determine their sex, breed, and age.

The clinical examination included assessments using the FAMACHA score and Dag score. Age classification relied on the presence of permanent teeth, categorizing animals as adult (1, 2, 3, 4, and >4 years) or young (<1 year) if nonpermanent teeth were observed. BCS was applied following Detweiler et al. [9], assessing the lumbar and sternum regions for classifications, including obese (1), fat (2), average (3), thin (4), and emaciated (5). FAMACHA scores were assigned based on conjunctival coloration, indicating nonanemia (1), mild (2), moderate (3), severe (4), or very severe anemia (5) [10]. The Dag score assessed fecal staining severity on the tail and crutch, categorized as normal (1), light (2), moderate (3), severe (4), or very severe (5) [11, 12]. The presence of bottle jaw was recorded. A total of 913 animals were randomly sampled for blood and fecal collection to detect GI parasites. The number of animals categorized by age and clinical examination is shown in Table 1.

### 2.2. Study Animals and Data Collection

The animals included in the study were over 1 month old and represented both sexes and various age groups. Prior to the investigation, all herds had refrained from anthelmintic treatment for at least 1 month. A total of 913 animals from 42 small-holder meat goat herds were included in the study. Each animal underwent blood and feces sample collection. A 5 ml blood sample was taken from the jugular vein and divided into 3 ml of serum and 2 ml of EDTA, which were stored in separate tubes. In addition, at least 10 g of fresh feces was collected from the rectum of each animal and placed in a zip-lock plastic bag. All samples were transported and stored at 4°C until laboratory diagnostics were conducted at the Faculty of Veterinary Medicine, Khon Kaen University. Data obtained from individual animals comprised signalment (including name/number, age, sex, and breed) along with findings from physical examinations (such as FAMACHA score, BCS, Dag score, and the presence of bottle jaw).

## 3. Laboratory Analysis

### 3.1. Blood Analysis

EDTA blood samples were processed to determine packed cell volume (PCV, %) by centrifuging EDTA in microhematocrit tubes at 3,000 rpm for 15 minutes. PCV (%) was calculated as the ratio of red blood cells to the total blood volume. Additionally, hemoglobin (Hb) levels (g/dL) were measured using the HemoCue (Hb 201+ System, manufactured by HemoCue AB, located at Kuvettgatan 1, SE-262 71, Ängelholm, Sweden).

Serum samples were assessed for the concentration of total serum protein (TP) (g/dL) using a hand refractometer (Erma, model D, Tokyo, Japan).

### 3.2. Fecal Analysis

#### 3.2.1. Fecal Egg Count and Identification of Worm Eggs

Fecal egg counts and the identification of worm eggs were conducted according to the methodology outlined by Brummaier et al. [13]. The subsequent differentiation of worm eggs based on their morphological characteristics followed the established protocol delineated by Taylor et al. [14].

#### 3.2.2. Coproculture and Larva Identification

Fecal samples from the same herd were pooled and cultured for larval identification following established protocols. At least 50 grams of fecal samples from each herd was combined in sterile plastic bags with an equal amount of sterile litter and moistened with distilled water. The pooled fecal samples were then incubated at an ambient temperature range of approximately 25–35°C for a period of 14 days within a dark environment. Following the incubation period, third-stage larvae were harvested using the modified Baermann technique over a 24-hour period. The larvae collected in a 15 ml conical tube were morphologically identified using a standard key. For identification purposes, hundreds of L3 larvae were fixed with 1–2% formalin and subjected to brief heating, facilitating morphological differentiation to determine the species or genus of gastrointestinal nematodes, as per the methodology described by Van Wyk et al. [15]. Notably, it should be acknowledged that certain nematode species, such as *Trichuris* spp., were not identified during this phase of the study.

#### 3.2.3. DNA Extraction, Genus-Specific PCRs, Amplification, and Sequencing

To ascertain the species infected with GI strongyle, pooled larval solutions from 41 herds in Khon Kaen were subjected to molecular techniques for nematode count confirmation. DNA extraction from larval pools was conducted using the Thermo Scientific GeneJET Genomic DNA Purification Kit (Thermo Fisher) as per the manufacturer's guidelines. DNA concentration was measured using a BioDrop DUO spectrophotometer, and the extracted DNA was stored at −20°C until further use. Genus-specific PCRs were employed to identify strongyle worms of the *Haemonchus* and *Trichostrongylus* genera in L3 samples, following the protocols outlined by Demeler et al. [16] and Mohammedsalih et al. [17]. *Haemonchus* spp. and *Trichostrongylus* spp. were identified based on larval morphology. PCRs targeted the amplification of partial internal transcribed spacer 2 (ITS-2) using specific primers designed for *Haemonchus* spp. and *Trichostrongylus* spp.

The PCR consisted of 12.5 *μ*l of DreamTaq Green PCR Master Mix (2X), 0.2 mM of each primer (Thermo Fisher Scientific, Waltham, USA), and 2 *μ*l (20–50 ng) of template DNA. Reaction conditions included an initial denaturation at 95°C for 3 min, followed by 40 cycles of denaturation at 95°C for 30 s, primer pair-specific annealing at the designated temperature for 30 s, and extension at 72°C for 30 s. A final elongation step at 72°C for 5 min was performed using a C1000 Touch Thermal Cycler (Bio-Rad, California, USA). Subsequently, PCR products were analyzed on 1.5% agarose gels stained with RedSafe (iNtRON Biotechnology, Korea). Purification of PCR products was carried out using the GF-1 AmbiClean Kit (Gel and PCR) (Vivantis, Malaysia), followed by submission to ATGC Co., Ltd (Pathum Thani, Thailand) for sequencing. Nucleotide sequences were compared against the NCBI database using the BLAST tool and aligned with published reference sequences using the BioEdit Sequence Alignment Editor. Preliminary sequencing was conducted on 10 PCR product samples.

### 3.3. Statistical Analysis

The data were analyzed using Microsoft Excel, Statistix software (version 8.0 for Windows), and MedCalc® version 22.021 (MedCalc Software Ltd., 2024). Descriptive statistics, including median, mean, standard deviation (SD), minimum (min), and maximum (max), were calculated for the parameters. Normal distribution was assessed using the Shapiro–Wilk test. Risk factors, including herd size, grazing practices, housing conditions, presence of other livestock, deworming programs, management protocols, and cleanliness of feed and water trays, were taken into consideration. A univariate analysis was performed to identify variables associated with positive herds and individual animals, with a significance threshold set at 0.05. Odds ratios, along with their corresponding 95% confidence intervals, were computed to gauge the strength of these associations.

Spearman rank correlations were employed to examine associations among age, BCS, FAMACHA score, Dag score, hematological parameters (PCV, Hb), biochemistry (total protein), and the number of GI parasite infections (EPG/OPG).

Statistical significance was defined as a *p* value less than 0.05 (^∗^ = *p* < 0.05; ^∗∗^ = *p* < 0.01; ^∗∗∗^ = *p* < 0.001). Most of the data did not conform to a normal distribution (*p* < 0.05, Shapiro–Wilk test), necessitating the use of nonparametric tests for statistical analysis.

### 3.4. Spatial Distribution

Quantum GIS was employed to generate a map illustrating the distribution of the cases.

## 4. Results

### 4.1. Prevalence and Spatial Distribution of Gastrointestinal Parasite Infections

#### 4.1.1. Microscopic Examination

**Figure 2:**
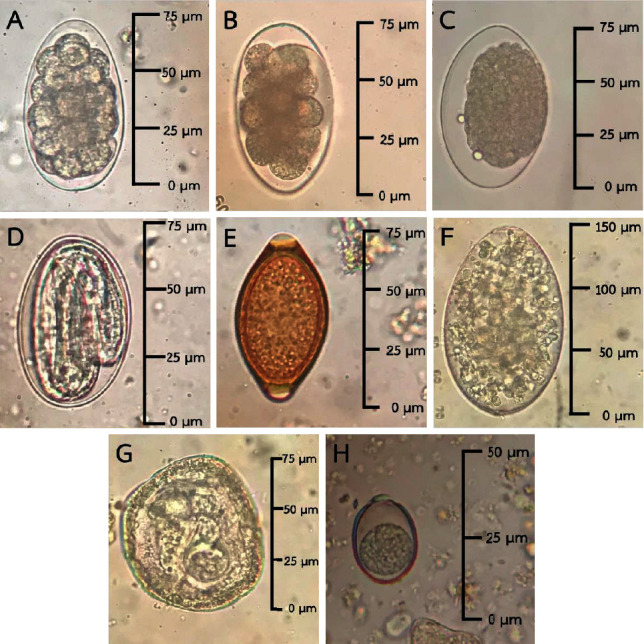
Morphology of GI parasite eggs or oocysts detected by microscopic examination. (A–C) Strongyle egg; (D) *Strongyloides* spp. (larvae egg); (E) *Trichuris* spp. egg; (F) *Paramphistomum* spp. egg; (G) *Moniezia* spp. egg; (H) *Eimeria* spp. oocyst (microscope image at 400x magnification).

GI parasites such as strongyle, *Strongyloides* spp., *Trichuris* spp., *Paramphistomum* spp. (rumen fluke), *Moniezia* spp., and *Eimeria* spp. oocysts were discovered in the herds (Figure 2). The number of eggs or oocysts from gastrointestinal parasite infections, categorized by age, is shown in Table 2.

#### 4.1.2. Seasonal Variations in the Prevalence of Gastrointestinal Parasite Infections

The prevalence of gastrointestinal parasite infections focuses on seasonal variations across districts. The data show fluctuations in parasite prevalence, with seasonal variations being significant. The wet season is associated with higher infection rates for various parasite species, including Strongyle, *Strongyloides* spp., *Paramphistomum* spp., *Moniezia* spp., and *Eimeria* spp., in different districts (Table 3). The prevalence of gastrointestinal parasites includes both single and mixed infections. Strongyle infection is the most common single parasite, accounting for over half of the cases, followed by *Trichuris* spp., *Moniezia* spp., and *Eimeria* spp. Mixed infections, while less frequent, involve various combinations of nematodes, cestodes, trematodes, and protozoa (Table 4). Notably, combinations of nematodes with protozoa make up a significant portion of mixed infections.

#### 4.1.3. Coproculture to Identify Strongyle Larva

**Figure 3:**
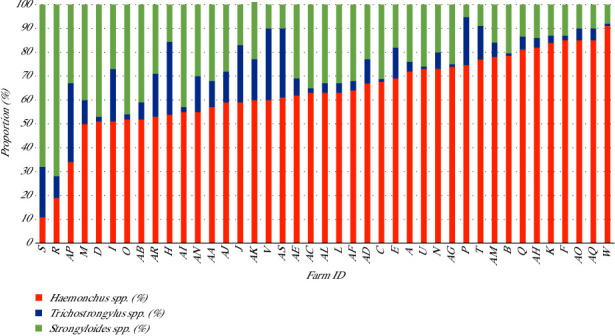
Proportion of strongyle larvae based on coproculture in 41 meat goat herds, Khon Kaen, Northeast Thailand.

In this study, the proportion of Strongyle larvae in each herd comprised *Haemonchus* spp., *Trichostrongylus* spp., and *Strongyloides* spp., as illustrated in Figure 3.

#### 4.1.4. Molecular Techniques for Identifying GI Strongyle Larva

**Figure 4:**
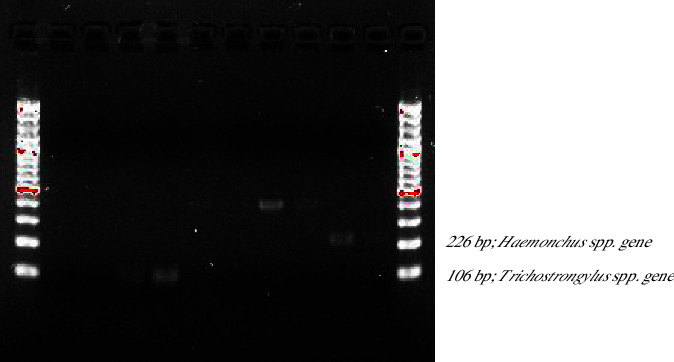
Restriction fragment of amplified ITS-2 regions of *Trichostrongylus* spp. (106 bp) and *Haemonchus* spp. (226 bp).

Ten samples yielded positive PCR results, comprising five samples of *Trichostrongylus* spp. and five samples of *Haemonchus* spp. The restriction sites of the ITS-2 region are depicted in Figure 4. Subsequently, all positive samples underwent further characterization through gene sequencing. Sequencing revealed that the larvae belonged to *H. contortus*, exhibiting 100% sequence identity and query cover with the GenBank database. Similarly, *Trichostrongylus* spp. demonstrated identical sequence characteristics to the GenBank database.

#### 4.1.5. Risk Factors Associated with GI Parasite Infection

The data indicate that herd management factors do not consistently exhibit significant associations with parasite prevalence across various parasite types (Table 5). However, there is a slight association observed between the frequency of cleaning feed and water trays and parasite prevalence, although it is not statistically significant.

GI parasite infection at animal levels examines the associations between variables, such as sex, age, and breed. Female goats have a significantly higher risk of strongyle infection. Older goats (over one year old) are also more prone to *Trichuris* spp., *Moniezia* spp., and *Eimeria* spp. infections, indicating vulnerability related to age. However, breed (Mixed Anglo-Nubian) does not demonstrate a significant correlation with parasite prevalence. The data are presented in Table 6.

#### 4.1.6. Association between Factors and Clinical Signs according to Laboratory Results

In this investigation, clinical signs were assessed using BCS, FAMACHA score, and Dag score. However, no sign of bottle jaw was detected. The results revealed significant differences between genders in certain aspects. Specifically, female subjects exhibited distinct variations in BCS (*p* < 0.001) and FAMACHA score (*p* < 0.01) compared to males, suggesting potential gender-related differences in these clinical signs. Additionally, significant variations were observed in packed cell volume (PCV) (*p* < 0.01), a key hematological parameter, indicating gender-related discrepancies in blood composition. However, no significant differences were found in other hematological parameters, such as hemoglobin levels (*p* > 0.05). Furthermore, biochemical analysis showed no significant disparities in total protein levels (*p* > 0.05) between female and male subjects. Notably, significant gender-related variations were identified in GI parasite infection rates, particularly for strongyle infections (*p* < 0.001). However, no significant differences were observed in the infection rates of other GI parasite species (*p* > 0.05). All gender-related parameters are presented in Table 7.

The clinical signs indicate that goats with poor BCS are more prone to increased anemia and fecal staining severity. Positive correlations were found between BCS and both FAMACHA score (*r* = 0.3, *p* < 0.01) and Dag score (*r* = 0.4, *p* < 0.001). There is also a positive correlation between FAMACHA score and Dag score (*r* = 0.29, *p* < 0.01) (Table 8). However, age does not appear to have a significant correlation with fecal staining severity.

Age shows moderate negative correlations with hematological parameters. Older goats may have lower PCV (*r* = −0.32, *p* < 0.01) and hemoglobin levels (*r* = −0.23, *p* < 0.05). In contrast, BCS and FAMACHA score exhibit strong negative correlations with hematological parameters. Goats with poorer BCS and higher FAMACHA score tend to have lower PCV (*r* = −0.45 to −0.48, *p* < 0.001) and hemoglobin levels (*r* = −0.47 to −0.55, *p* < 0.001). The Dag score also displays moderate negative correlations with hematological parameters. Goats with more severe fecal staining may have lower PCV (*r* = −0.34, *p* < 0.01) and hemoglobin levels (*r* = −0.39, *p* < 0.001). Additionally, total protein shows a moderate positive correlation with age (*r* = 0.38, *p* < 0.001), indicating that older goats may have higher total protein levels.

In terms of GI parasite infections in meat goats, BCS shows a positive correlation with strongyle (*r* = 0.3, *p* < 0.01) and FAMACHA scores (*r* = 0.33, *p* < 0.01). Moreover, *Strongyloides* spp. displays a positive correlation with FAMACHA score (*r* = 0.23, *p* < 0.05) and Dag score (*r* = 0.41, *p* < 0.001). The Dag score also demonstrates positive correlations with strongyle and *Strongyloides* spp. Additionally, *Eimeria* spp. shows a positive correlation with FAMACHA score (*r* = 0.28, *p* < 0.01) and Dag score (*r* = 0.37, *p* < 0.001).

Regarding GI parasite infections, significant positive correlations were detected between strongyle infections and BCS (*r* = 0.30, *p* < 0.01) and FAMACHA score (*r* = 0.33, *p* < 0.01). Moreover, *Strongyloides* spp. infections exhibited significant positive correlations with age, FAMACHA score, and Dag score.

The correlation of various parameters, including PCV (packed cell volume), hemoglobin, and total protein, with different gastrointestinal (GI) parasite infections are presented in Table 9. PCV and hemoglobin exhibit significant negative correlations with strongyle EPG counts (*r* = −0.38, *p* < 0.001, and *r* = −0.44, *p* < 0.001, respectively). Additionally, hemoglobin and total protein display significant negative correlations with *Strongyloides* spp. EPG counts (*r* = −0.22, *p* < 0.05, and *r* = −0.25, *p* < 0.05, respectively). Furthermore, hemoglobin (*r* = −0.31, *p* < 0.01) and total protein (*r* = −0.38, *p* < 0.001) demonstrate a moderate negative correlation with *Eimeria* spp. counts.

## 5. Discussions

Infection by GI parasites was generally found in meat goat herds in Khon Kaen, Northeast Thailand. All herds had strongyle nematodes, with *Trichuris* spp., *Moniezia* spp., and *Eimeria* spp. occurring in approximately 60–70% of herds. Coinfection involving at least two types of nematodes, such as Strongyle and *Strongyloides* spp., Strongyle and *Trichuris* spp., or *Strongyloides* spp. and *Trichuris* spp., as well as cestodes, trematodes, or protozoa, was observed in 90% of herds. This finding is consistent with previous studies that showed a high prevalence of GI parasitic infections in herds and common coinfections of nematodes, protozoa, and cestodes in small ruminants [18–20]. In this study, a few herds showed trematode infections, such as *Paramphistomum* spp., which could be attributed to the geographical location of these herds near natural water resources, such as floating swamps, streams, flood plains, or rivers. These areas are habitats for living snails, which are intermediate hosts of trematodes [21]. Additionally, neighboring beef cattle herds that share pasture with meat goats may transmit trematodes to other species.

The spatial distribution of GI parasitic infections in meat goat herds can provide insights into the main helminth infections in this area. *Haemonchus* spp., *Trichostrongylus* spp., *Trichuris* spp., and *Strongyloides* spp. were dominant nematodes in meat goats. Molecular techniques confirmed that the dominant *Haemonchus* spp. was *H. contortus,* which was highly related to *H. contortus* strains in Kanchanaburi, Thailand (access no. MT294437.1) [22], Cameroon (no. MN708986.1) [19], and Nigeria (no. LC368075.1) [23] (100% sequence identity and 100% query cover to GenBank database). *Trichostrongylus* spp. were closely related to *T. colubriformis* strains in Kanchanaburi, Thailand (no. MT294439.1) [22] and Egypt (no. MK936884.1) [24], as well as *T. axei* strains in Europe (no. ON677955.1) [25] and Ghana (no. MH481571.1) [26] (100% sequence identity and 100% query cover to GenBank database). Similarly, cestode and protozoa infections were also common health problems in meat goats in this area.

This study revealed an association between GI strongyle and *Eimeria* spp. infections during the wet season, consistent with prior research indicating a propensity for elevated parasite infection rates in livestock during wet conditions, as evidenced in studies conducted in Senegal and India [27, 28]. Conversely, GI nematode burdens may surge during the dry season in the West Indian Islands [29], suggesting geographical variations in GI parasite prevalence among ruminants.

Laboratory analyses found that male animals had higher body condition scores (BCS) and FAMACHA score than females in terms of the association between factors and clinical signs. This supports previous studies, indicating that females are more susceptible to strongyle infections [5, 30]. Female goats, in particular, are vulnerable to parasitic infections due to their reproductive cycles, which affect the secretion of worm eggs. This increased susceptibility is most evident during the periparturient and postparturient periods, characterized by heightened physiological stress and compromised immunity, resulting in greater shedding of strongyle eggs [30–32].

Concerning age factors, young animals exhibited a stronger association with strongyle infection, whereas older animals displayed a higher prevalence of *Trichuris* spp., *Moniezia* spp., and *Eimeria* spp. infections, consistent with the previous literature [33, 34].

An escalation in strongyle eggs corresponded with clinical manifestations, such as anemia, diarrhea, emaciation, and bottle jaw, indicative of heightened adult worm infections. This corroborates earlier studies, demonstrating that nematodes, particularly *H. contortus* infections, can provoke progressive anemia and hypoproteinemia in small ruminants [10, 14, 35–37]. In critical scenarios, such as instances of poor BCS, elevated FAMACHA or Dag scores, or markedly low levels of PCV or Hb, progressive anemia can precipitate rapid mortality, especially when compounded by inadequate nutrition or stress [14, 38]. Additionally, poor BCS was identified as a risk factor for GI strongyle infections in livestock animals [39].

Based on our investigation, a notable prevalence of strongyle nematode infections was identified within meat goat herds. Premunition, characterized by early-life exposure leading to subsequent resistance to further infection, may manifest within these herds, with chronic infections persisting if the infecting strongyle remains within the host [38]. Notably, in cases where hosts do not exhibit clinical signs, such as anemia, progressive weight loss, diarrhea, or bottle jaw, treatment may not be warranted for this infective condition. To facilitate targeted selective treatments, farmers could employ the FAMACHA score to assess subclinical strongyle infections, with scores of 1–2 indicating no immediate requirement for anthelmintic treatment, while scores of 3 close to 4 or 4–5 signify an urgent need for intervention. In our area, BCS was not employed to guide treatment decisions due to prevalent undernutrition, with a majority of adult animals presenting as thin or emaciated. Moreover, animals experiencing malnutrition are predisposed to heightened risks of GI parasite infections owing to compromised immune responses [39–41]. We advocate for treating animals exhibiting poor BCS alongside FAMACHA scores of 3 to 4 and Dag scores of 2. However, it is crucial to acknowledge that the elimination of stable and established infections may lead to the loss of premunition, potentially resulting in rapid reinfection, with an increased parasite burden [38, 42]. The scope of this study was confined to 42 small-holder meat goat herds situated in an agricultural area known for its prevalence of meat goat farming in Khon Kaen, Thailand. While univariate analysis revealed significant variations in risk factors related to GI parasite infection, such as season, gender, and age, deeper exploration and intervention may be required to fully understand their implications. Future research initiatives are warranted to assess the effectiveness of pharmaceutical interventions and to evaluate the emergence of antiparasitic drug resistance in GI parasitic infections.

## 6. Conclusions

In conclusion, gastrointestinal (GI) parasite infections are common among meat goats in Khon Kaen, Northeast Thailand. Strongyle nematodes emerged as the predominant parasites in all herds, with coinfections involving multiple parasite types being commonplace, and seasonal variations, with wet seasons correlating with increased infections of strongyle and *Eimeria* spp. Moreover, gender and age were identified as significant factors influencing susceptibility to GI parasite infection. Clinical indicators such as anemia, diarrhea, and emaciation were found to be associated with strongyle infections. The findings underscore the necessity for implementing effective management strategies targeting animals with poor BCS and elevated FAMACHA scores to mitigate the impact of GI parasitic infections on meat goat health and productivity.

## Figures and Tables

**Table 1:** Distribution of animals categorized by age and clinical examination.

Age (yr)	Total	BCS							FAMACHA score									Dag score				
		2	2.5	3	3.5	4	4.5	5	1	1.5	2	2.5	3	3.5	4	4.5	5	1	2	3	4	5
<1	274	1	1	130	101	39	2	—	18	1	108	1	130	5	8	—	3	254	14	3	3	—
1	168	—	—	42	82	41	3	—	4	—	53	1	103	3	2	1	1	159	8	—	—	1
2	92	—	—	21	42	26	2	1	2	—	26	1	59	2	1	—	1	83	8	1	—	—
3	139	—	1	41	50	41	6	—	1	—	32	—	97	4	5	—	—	134	4	1	—	—
4	118	1	4	24	47	34	8	—	—	—	14	1	94	4	3	2	—	108	9	—	1	—
>4	122	—	2	12	36	57	13	2	—	—	18	1	85	8	8	1	1	112	9	—	—	1

Total	913	2	8	270	358	238	34	3	25	1	251	5	568	26	27	4	6	850	52	5	4	2
	(%)	0.22	0.88	29.57	39.21	26.07	3.72	0.33	2.74	0.11	27.49	0.55	62.21	2.85	2.96	0.44	0.66	93.10	5.70	0.55	0.44	0.22

*N* = number of animals.

**Table 2:** The distribution of eggs or oocysts from gastrointestinal parasite infections, stratified by age.

Age (yr)	*N*	Strongyle (EPG)		*Strongyloides* spp. (EPG)		*Trichuris* spp. (EPG)		*Paramphistomum* spp. (EPG)		*Moniezia* spp. (EPG)		*Eimeria* spp. (OPG)	
		Mean ± SD	95% CI	Mean ± SD	95% CI	Mean ± SD	95% CI	Mean ± SD	95% CI	Mean ± SD	95% CI	Mean ± SD	95% CI
<1	274	75.5 ± 215.4	49.9–101.1	1.2 ± 9.9	0–2.4	3.6 ± 13.2	2.0–5.1	0.04 ± 0.7	0–0.1	30.9 ± 174.5	10.1–51.7	164.7 ± 1,731.0	0–370.6
1	168	124.1 ± 413.8	61.1–187.2	1.3 ± 7.1	0.2–2.4	5.9 ± 19.2	3.0–8.9	—	—	15.0 ± 121.2	0–33.4	7.1 ± 29.0	2.7–11.6
2	92	158.7 ± 480.0	59.2–258.2	2.2 ± 9.6	0.2–4.2	3.2 ± 10.9	0.9–5.4	—	—	2.4 ± 11.1	0.1–4.7	11.0 ± 46.5	1.4–20.7
3	139	53.97 ± 100.2	37.1–70.7	0.1 ± 1.2	0–0.3	1.1 ± 4.7	0.3–1.9	—	—	3.5 ± 26.4	0–8.0	26.0 ± 255.3	0–68.9
4	118	62.6 ± 91.6	45.9–79.3	—	—	2.7 ± 8.2	1.2–4.3	0.03 ± 0.3	0–0.1	1.5 ± 8.7	0–3.1	4.1 ± 27.8	0–9.2
>4	122	114.1 ± 282.4	63.4–164.7	1.1 ± 4.4	0.3–1.9	1.4 ± 5.0	0.5–2.3	0.1 ± 0.9	0–0.3	2.8 ± 21.0	0–6.6	14.8 ± 111.4	0–34.8

**Table 3:** Seasonal fluctuations in the prevalence of gastrointestinal parasite infections among meat goat herds in Khon Kaen, Thailand.

District	Farm	Strongyle (%)		*Strongyloides* spp. (%)		*Trichuris* spp. (%)		*Paramphistomum* spp. (%)		*Moniezia* spp. (%)		*Eimeria* spp. (%)	
		Dry	Wet	Dry	Wet	Dry	Wet	Dry	Wet	Dry	Wet	Dry	Wet
Ban Fang	A	35.7	54.5	0.0	4.5	14.3	9.1	0.0	0.0	7.1	9.1	14.3	4.5
	B	55.6	ND	0.0	ND	0.0	ND	0.0	ND	0.0	ND	0.0	ND
	C	66.7	ND	0.0	ND	0.0	ND	0.0	ND	0.0	ND	11.1	ND

Ban Haet	D	50.0	63.3	0.0	0.0	5.6	0.0	0.0	0.0	0.0	0.0	5.6	22.4

Muang	E	ND	73.9	ND	0.0	ND	17.4	ND	0.0	ND	0.0	ND	8.7
	F	94.4	ND	0.0	ND	44.4	ND	0.0	ND	11.1	ND	11.1	ND

Nam Phong	G	20.0	ND	0.0	ND	0.0	ND	0.0	ND	0.0	ND	0.0	ND
	H	100.0	ND	9.5	ND	23.8	ND	0.0	ND	33.3	ND	4.8	ND

Nong Ruea	I	38.9	ND	0.0	ND	16.7	ND	0.0	ND	0.0	ND	5.6	ND
	J	42.9	ND	0.0	ND	28.6	ND	0.0	ND	0.0	ND	28.6	ND
	K	88.9	82.4	0.0	0.0	22.2	5.9	0.0	0.0	11.1	5.9	11.1	5.9
	L	78.1	91.7	0.0	0.0	15.6	8.3	0.0	0.0	12.5	12.5	9.4	16.7
	M	57.1	83.3	14.3	8.3	42.9	25.0	0.0	0.0	0.0	0.0	28.6	8.3
	N	100.0	ND	0.0	ND	50.0	ND	0.0	ND	25.0	ND	0.0	ND
	O	22.2	ND	0.0	ND	0.0	ND	0.0	ND	11.1	ND	0.0	ND
	P	100.0	ND	0.0	ND	0.0	ND	0.0	ND	0.0	ND	0.0	ND
	Q	75.0	ND	0.0	ND	37.5	ND	0.0	ND	0.0	ND	0.0	ND

Phu Wiang	R	28.6	ND	14.3	ND	14.3	ND	0.0	ND	14.3	ND	0.0	ND
	S	20.0	ND	0.0	ND	0.0	ND	0.0	ND	0.0	ND	0.0	ND
	T	100.0	81.8	0.0	0.0	40.0	9.1	0.0	0.0	20.0	0.0	0.0	0.0
	U	46.7	20.0	0.0	0.0	26.7	20.0	0.0	0.0	0.0	6.7	13.3	26.7
	V	71.4	66.7	42.9	8.3	14.3	8.3	0.0	0.0	0.0	0.0	14.3	0.0

Si Chomphu	W	ND	90.0	ND	0.0	ND	5.0	ND	0.0	ND	30.0	ND	25.0
	AA	ND	82.4	ND	0.0	ND	5.9	ND	5.9	ND	5.9	ND	0.0
	AB	ND	90.9	ND	36.4	ND	18.2	ND	0.0	ND	0.0	ND	0.0
	AC	ND	93.8	ND	0.0	ND	12.5	ND	0.0	ND	6.3	ND	0.0
	AD	ND	85.3	ND	17.6	ND	44.1	ND	0.0	ND	2.9	ND	26.5
	AE	ND	100.0	ND	18.2	ND	45.5	ND	0.0	ND	9.1	ND	72.7
	AF	83.3	85.7	41.7	32.1	16.7	60.7	0.0	0.0	50.0	0.0	83.3	32.1
	AG	ND	72.7	ND	0.0	ND	0.0	ND	0.0	ND	9.1	ND	0.0
	AH	ND	92.3	ND	0.0	ND	46.2	ND	0.0	ND	0.0	ND	7.7
	AI	ND	69.2	ND	0.0	ND	0.0	ND	0.0	ND	3.8	ND	84.6
	AJ	ND	70.0	ND	30.0	ND	30.0	ND	0.0	ND	10.0	ND	60.0
	AK	ND	89.3	ND	0.0	ND	0.0	ND	0.0	ND	7.1	ND	0.0
	AL	ND	84.6	ND	0.0	ND	15.4	ND	0.0	ND	3.8	ND	7.7

Wiang Kao	AM	57.1	84.0	0.0	4.0	14.3	28.0	0.0	0.0	14.3	16.0	0.0	12.0
	AN	75.0	95.7	16.7	0.0	8.3	8.7	0.0	0.0	0.0	4.3	0.0	26.1
	AO	100.0	81.0	0.0	9.5	28.6	9.5	0.0	0.0	14.3	14.3	0.0	4.8
	AP	100.0	ND	9.1	ND	0.0	ND	27.3	ND	18.2	ND	9.1	ND
	AQ	100.0	96.3	0.0	7.4	0.0	11.1	0.0	3.7	9.1	11.1	9.1	51.9
	AR	ND	69.6	ND	0.0	ND	26.1	ND	0.0	ND	13.0	ND	8.7
	AS	ND	100.0	ND	9.1	ND	0.0	ND	0.0	ND	0.0	ND	36.4

Total (%)	42	27 (100.0)	28 (100.0)	7 (25.9)	12 (42.8)	19 (70.3)	23 (82.1)	1 (3.7)	2 (7.1)	14 (51.8)	19 (67.8)	15 (55.5)	21 (75.0)

ND = Not done.

**Table 4:** Characteristics of gastrointestinal (GI) parasite infection as determined by the fecal egg count technique.

Characteristic	Parasites	Number positive (%)
Single	Strongyle	417 (55.67)
	*Strongyloides* spp.	1 (0.13)
	*Trichuris* spp.	15 (2.00)
	*Moniezia* spp.	7 (0.93)
	*Eimeria* spp.	19 (2.54)

Mixed	2-Type nematode	
	(i) Strongyle and *Strongyloides* spp.	14 (1.87)
	(ii) Strongyle and *Trichuris* spp.	81 (10.81)
	(iii) *Strongyloides* spp. and *Trichuris* spp.	1 (0.13)
	3-Type nematode	
	(i) Strongyle, *Strongyloides* spp., and *Trichuris* spp.	10 (1.34)
	Nematode and Cestode	
	(i) Strongyle and *Moniezia* spp.	32
	(ii) Strongyle, *Strongyloides* spp., and *Moniezia* spp.	1 (0.13)
	(iii) Strongyle, *Trichuris* spp., and *Moniezia* spp.	10 (1.34)
	(iv) *Trichuris* spp. and *Moniezia* spp.	2 (0.27)
	Nematode and Trematode	
	(i) Strongyle and *Paramphistomum* spp.	3 (0.40)
	(ii) Strongyle, *Strongyloides* spp., and *Paramphistomum* spp.	1 (0.13)
	Nematode and Protozoa	
	(i) Strongyle and *Eimeria* spp.	83 (11.08)
	(ii) *Trichuris* spp. and *Eimeria* spp.	2 (0.27)
	(iii) Strongyle, *Strongyloides* spp., and *Eimeria* spp.	10 (1.34)
	(iv) Strongyle, *Trichuris* spp., and *Eimeria* spp.	15 (2.00)
	(v) Strongyle, *Strongyloides* spp., *Trichuris* spp., and *Eimeria* spp.	9 (1.20)
	Cestode and Protozoa	
	(i) *Moniezia* spp. and *Eimeria* spp.	1 (0.13)
	Nematode, Cestode, and Protozoa	
	(i) Strongyle, *Moniezia* spp., and *Eimeria* spp.	9 (1.20)
	(ii) *Strongyloides* spp., *Moniezia* spp., and *Eimeria* spp.	1 (0.13)
	(iii) Strongyle, *Trichuris* spp., *Moniezia* spp., and *Eimeria* spp.	3 (0.40)
	(iv) Strongyle, *Strongyloides* spp., *Trichuris* spp., *Moniezia* spp., and *Eimeria* spp.	1 (0.13)
	Nematode, Cestode, and Trematode	
	(i) Strongyle, *Moniezia* spp., and *Paramphistomum* spp.	1 (0.13)
	Nematode and Trematode	
	(i) Strongyle and *Paramphistomum* spp.	3 (0.40)
	(ii) Strongyle and *Strongyloides* spp. and *Paramphistomum* spp.	1 (0.13)

**Table 5:** Univariate analysis of GI parasite infection in meat goat herds at the herd level.

GI parasites	Factors	Category	OR	95% CI	*p* value
Nematodes	Herd size	≥33	0.7	0.01–36.25	0.9
Trematodes	Herd size	≥33	3.2	0.26–38.42	0.4
Cestodes	Herd size	≥33	2.6	0.64–10.05	0.2
Protozoa	Herd size	≥33	3.7	0.83–16.04	0.1
Nematodes	Grazing	Communal	0.1	0.002–6.64	0.3
Trematodes	Grazing	Communal	6.0	0.41–86.97	0.2
Cestodes	Grazing	Communal	5.9	0.29–118.20	0.2
Protozoa	Grazing	Communal	0.5	0.05–3.67	0.5
Nematodes	Housing	Loose stalls	0.2	0.003–11.51	0.4
Trematodes	Housing	Loose stalls	0.6	0.02–13.30	0.8
Cestodes	Housing	Loose stalls	0.7	0.13–3.62	0.7
Protozoa	Housing	Loose stalls	1.3	0.21–7.75	0.8
Nematodes	Other livestock	Presence	0.5	0.01–24.30	0.7
Trematodes	Other livestock	Presence	5.1	0.41–62.00	0.2
Cestodes	Other livestock	Presence	0.5	0.13–2.01	0.3
Protozoa	Other livestock	Presence	0.7	0.18–2.82	0.6
Nematodes	Deworming program	Not have	0.2	0.004–13.33	0.5
Trematodes	Deworming program	Not have	0.6	0.02–13.30	0.8
Cestodes	Deworming program	Not have	0.7	0.13–3.62	0.7
Nematodes	Protocol deworming	Individual animal	0.7	0.01–36.25	0.9
Trematodes	Protocol deworming	Individual animal	3.2	0.26–38.42	0.4
Cestodes	Protocol deworming	Individual animal	2.6	0.64–10.05	0.2
Nematodes	Grouping of animals	Mixed age	2.7	0.05–146.25	0.6
Trematodes	Grouping of animals	Mixed age	0.7	0.05–8.45	0.8
Cestodes	Grouping of animals	Mixed age	1.8	0.42–7.13	0.4
Protozoa	Grouping of animals	Mixed age	1.2	0.28–5.06	0.8
Nematodes	Roughage management	Pasture paddock	0.5	0.01–24.30	0.7
Trematodes	Roughage management	Pasture paddock	1.1	0.09–13.63	0.9
Cestodes	Roughage management	Pasture paddock	2.4	0.53–10.40	0.3
Protozoa	Roughage management	Pasture paddock	0.7	0.18–2.82	0.6
Nematodes	Rotate pasture	No	5.6	0.10–308.99	0.4
Trematodes	Rotate pasture	No	0.3	0.02–3.87	0.4
Cestodes	Rotate pasture	No	0.9	0.14–5.50	0.9
Protozoa	Rotate pasture	No	1.0	0.15–6.25	1.0
Nematodes	Position conc. feed tray	Ground	0.1	0.002–8.17	0.3
Trematodes	Position conc. feed tray	Ground	0.9	0.04–19.82	0.9
Cestodes	Position conc. feed tray	Ground	0.8	0.12–5.49	0.8
Protozoa	Position conc. feed tray	Ground	0.7	0.10–4.89	0.7
Nematodes	Position roughage tray	Ground	0.1	0.001–5.18	0.2
Trematodes	Position roughage tray	Ground	1.5	0.06–35.10	0.8
Cestodes	Position roughage tray	Ground	1.1	0.09–13.48	0.9
Protozoa	Position roughage tray	Ground	0.2	0.01–2.69	0.2
Nematodes	Feces contaminated in feed or water tray	Found	0.3	0.01–17.31	0.6
Trematodes	Feces contaminated in feed or water tray	Found	19.7	0.93–413.52	0.1
Cestodes	Feces contaminated in feed or water tray	Found	2.7	0.49–15.01	0.2
Protozoa	Feces contaminated in feed or water tray	Found	6.2	0.69–54.64	0.1
Nematodes	Frequency of cleaning feed and water tray	2–3 days/time	0.6	0.01–29.83	0.8
Trematodes	Frequency of cleaning feed and water tray	2–3 days/time	0.9	0.07–10.74	0.9
Cestodes	Frequency of cleaning feed and water tray	2–3 days/time	0.8	0.20–2.77	0.7
Protozoa	Frequency of cleaning feed and water tray	2–3 days/time	0.6	0.16–2.37	0.5

**Table 6:** Univariate analysis of GI parasite infection at the animal level.

GI parasites	Factors	Category	Number positive (%)	OR	95% CI	*p* value
Strongyle	Season	Wet	700 (76.7)	1.81	1.32–2.48	**<0.001**
*Strongyloides* spp.	Season	Wet	49 (5.4)	1.2	0.64–2.23	0.57
*Trichuris* spp.	Season	Wet	149 (16.3)	1.05	0.72–1.51	0.81
*Paramphistomum* spp.	Season	Wet	5 (0.5)	0.35	0.05–2.08	0.25
*Moniezia* spp.	Season	Wet	68 (7.4)	0.64	0.38–1.05	0.08
*Eimeria* spp.	Season	Wet	153 (16.8)	2.04	1.35–3.07	**<0.001**
Strongyle	Sex	Female	700 (76.7)	2.76	1.75–4.34	**<0.0001**
*Strongyloides* spp.	Sex	Female	49 (5.4)	2.63	0.62–11.02	0.19
*Trichuris* spp.	Sex	Female	149 (16.3)	1.43	0.73–2.75	0.29
*Paramphistomum* spp.	Sex	Female	5 (0.5)	1.2	0.06–21.90	0.9
*Moniezia* spp.	Sex	Female	68 (7.4)	0.8	0.36–1.72	0.56
*Eimeria* spp.	Sex	Female	153 (16.8)	0.77	0.44–1.33	0.36
Strongyle	Age	<1 year	700 (76.7)	1.41	1.03–1.91	**<0.05**
*Strongyloides* spp.	Age	>1 year	49 (5.4)	1.22	0.68–2.16	0.5
*Trichuris* spp.	Age	>1 year	149 (16.3)	1.91	1.33–2.73	**<0.001**
*Paramphistomum* spp.	Age	>1 year	5 (0.5)	0.26	0.02–2.37	0.24
*Moniezia* spp.	Age	>1 year	68 (7.4)	1.92	1.15–3.20	**<0.05**
*Eimeria* spp.	Age	>1 year	153 (16.8)	1.95	1.36–2.78	**<0.001**
Strongyle	Breed	Mixed Anglo-Nubian	700 (76.7)	1.04	0.69–1.56	0.83
*Strongyloides* spp.	Breed	Mixed Anglo-Nubian	49 (5.4)	0.63	0.26–1.50	0.3
*Trichuris* spp.	Breed	Mixed Anglo-Nubian	149 (16.3)	0.72	0.43–1.18	0.19
*Paramphistomum* spp.	Breed	Mixed Anglo-Nubian	5 (0.5)	1.15	0.12–10.36	0.9
*Moniezia* spp.	Breed	Mixed Anglo-Nubian	68 (7.4)	0.59	0.27–1.26	0.18
*Eimeria* spp.	Breed	Mixed Anglo-Nubian	153 (16.8)	0.93	0.58–1.47	0.76

Bold value shows that significant association was classified as *p* < 0.05.

**Table 7:** Wilcoxon test results comparing BCS, FAMACHA score, Dag score, blood parameters, and fecal analysis outcomes between male and female groups (*n* = 913).

Parameter	Female (mean (N))	Male (mean (N))	*p* value
Clinical sign			
BCS	476.9 (824)	273.0 (89)	^∗∗∗^
FAMACHA score	464.6 (824)	387.0 (89)	^∗∗^
Dag score	458.1 (824)	446.8 (89)	NS
Hematology			
PCV (%)	411.4 (751)	493.1 (88)	^∗∗^
Hemoglobin (g/dL)	39.7 (69)	50.8 (13)	NS
Biochemistry			
Total protein (g/dL)	426.1 (761)	420.0 (89)	NS
GI parasites			
Strongyle (EPG)	469.4 (824)	342.4 (89)	^∗∗∗^
*Strongyloides* spp. (EPG)	458.5 (824)	443.1 (89)	NS
*Trichuris* spp. (EPG)	458.9 (824)	439.1 (89)	NS
*Paramphistomum* spp. (EPG)	457.3 (824)	454.5 (89)	NS
*Moniezia* spp. (EPG)	456.3 (824)	463.6 (89)	NS
*Eimeria* spp. (OPG)	454.8 (824)	477.0 (89)	NS

^∗^ = *p* < 0.05; ^∗∗^ = *p* < 0.01; ^∗∗∗^ = *p* < 0.001; NS = not significant.

**Table 8:** Spearman's correlation analysis for age, BCS, FAMACHA score, and Dag score in relation to clinical signs, blood parameters, and fecal analysis outcomes (*n* = 913).

Parameter	Age		BCS		FAMACHA score		Dag score	
	*r*=	*p* value	*r*=	*p* value	*r*=	*p* value	*r*=	*p* value
Clinical sign								
BCS	0.27	^∗^	—	—	0.30	^∗∗^	0.40	^∗∗∗^
FAMACHA score	0.55	NS	0.30	^∗∗^	—	—	0.29	^∗∗^
Dag score	−0.08	NS	0.40	^∗∗∗^	0.29	^∗∗^	—	—
Hematology								
PCV (%)	−0.32	^∗∗^	−0.45	^∗∗∗^	−0.48	^∗∗∗^	−0.34	^∗∗^
Hemoglobin (g/dL)	−0.23	^∗^	−0.47	^∗∗∗^	−0.55	^∗∗∗^	−0.39	^∗∗∗^
Biochemistry								
Total protein (g/dL)	0.38	^∗∗∗^	−0.05	NS	−0.19	NS	−0.21	NS
GI parasites								
Strongyle (EPG)	0.12	NS	0.30	^∗∗^	0.33	^∗∗^	0.26	^∗^
*Strongyloides* spp. (EPG)	−0.17	NS	0.15	NS	0.23	^∗^	0.41	^∗∗∗^
*Trichuris* spp. (EPG)	−0.09	NS	0.12	NS	0.05	NS	0.15	NS
*Moniezia* spp. (EPG)	−0.15	NS	0.12	NS	0.07	NS	0.18	NS
*Eimeria* spp. (OPG)	−0.21	NS	−0.07	NS	0.28	^∗∗^	0.37	^∗∗∗^

^∗^ = *p* < 0.05; ^∗∗^ = *p* < 0.01; ^∗∗∗^ = *p* < 0.001; NS = not significant.

**Table 9:** Results of Spearman's correlation for PCV, hemoglobin, and total protein according to GI parasite infection (*n* = 913).

Parameter	PCV		Hemoglobin		Total protein	
	*r*=	*p* value	*r*=	*p* value	*r*=	*p* value
GI parasites						
Strongyle (EPG)	−0.38	^∗∗∗^	−0.44	^∗∗∗^	−0.15	NS
*Strongyloides* spp. (EPG)	−0.05	NS	−0.22	^∗^	−0.25	^∗^
*Trichuris* spp. (EPG)	−0.15	NS	−0.10	NS	−0.05	NS
*Moniezia* spp. (EPG)	0.001	NS	−0.14	NS	−0.10	NS
*Eimeria* spp. (OPG)	−0.08	NS	−0.31	^∗∗^	−0.38	^∗∗∗^

^∗^ = *p* < 0.05; ^∗∗^ = *p* < 0.01; ^∗∗∗^ = *p* < 0.001; NS = not significant.

## Data Availability

The datasets used and analyzed in this study are available from the corresponding author upon reasonable request.
